# Quality of Service-Aware Multi-Objective Enhanced Differential Evolution Optimization for Time Slotted Channel Hopping Scheduling in Heterogeneous Internet of Things Sensor Networks

**DOI:** 10.3390/s24185987

**Published:** 2024-09-15

**Authors:** Aida Vatankhah, Ramiro Liscano

**Affiliations:** Department of Electrical, Computer and Software Engineering, Ontario Tech University, Oshawa, ON L1G 0C5, Canada; ramiro.liscano@ontariotechu.ca

**Keywords:** Internet of Things, IEEE 802.15.4 TSCH schedule, differential evolution optimization, quality of service

## Abstract

The emergence of the Internet of Things (IoT) has attracted significant attention in industrial environments. These applications necessitate meeting stringent latency and reliability standards. To address this, the IEEE 802.15.4e standard introduces a novel Medium Access Control (MAC) protocol called Time Slotted Channel Hopping (TSCH). Designing a centralized scheduling system that simultaneously achieves the required Quality of Service (QoS) is challenging due to the multi-objective optimization nature of the problem. This paper introduces a novel optimization algorithm, QoS-aware Multi-objective enhanced Differential Evolution optimization (QMDE), designed to handle the QoS metrics, such as delay and packet loss, across multiple services in heterogeneous networks while also achieving the anticipated service throughput. Through co-simulation between TSCH-SIM and Matlab, R2023a we conducted multiple simulations across diverse sensor network topologies and industrial QoS scenarios. The evaluation results illustrate that an optimal schedule generated by QMDE can effectively fulfill the QoS requirements of closed-loop supervisory control and condition monitoring industrial services in sensor networks from 16 to 100 nodes. Through extensive simulations and comparative evaluations against the Traffic-Aware Scheduling Algorithm (TASA), this study reveals the superior performance of QMDE, achieving significant enhancements in both Packet Delivery Ratio (PDR) and delay metrics.

## 1. Introduction

In the context of Industry 4.0, the convergence of advanced technologies, particularly the integration of the Internet of Things (IoT), has revolutionized industrial processes. This paradigm shift emphasizes how devices work together smartly, enabling seamless communication and data exchange throughout the entire manufacturing ecosystem. Achieving the Industry 4.0 vision requires significant transformations in wireless sensor network design to support complex machine communications under stringent Quality of Service (QoS) requirements alongside an increasing number of sensors that monitor and report sensory data. A pervasive wireless sensor network (WSN) topology must cover all sensors across the environment, enabling data transmission to a central sink node. This node aggregates and processes information through automated, optimized routines to streamline industrial operations [1].

Within the domain of IoT networks, one of the key elements contributing to the connectivity of industrial wireless networks is the Time Slotted Channel Hopping (TSCH) mechanism, which stems from the IEEE 802.15.4 amendment [2,3]; however, the standard does not define how TSCH packet transmission schedule is defined.

Efficiently scheduling transmissions in industrial wireless networks is crucial for optimizing network performance and resource utilization. A well-designed schedule ensures that data transmissions occur in a timely manner, minimizing collisions, latency, and packet loss. Industrial sensor networks in particular face the challenge of accommodating sensors with different packet rates and QoS requirements, making it difficult to determine an optimal transmission schedule.

Existing studies primarily focus on optimizing specific metrics within the scope of a single application, often overlooking the unique QoS requirements of each application and the common scenario of multiple applications operating concurrently on the same network. Rather than merely minimizing or maximizing a metric, each application must meet its distinct QoS demands. Furthermore, many studies assume fixed packet rates, disregarding the heterogeneity of packet rates typical in industrial environments. The objective of this paper is to develop a TSCH scheduling algorithm capable of addressing these diverse QoS requirements across multiple applications within a wireless sensor network. To the best of our knowledge, this approach marks the first attempt to introduce the simultaneous consideration of heterogeneous application specifications, particularly in terms of packet rates and the challenge of meeting the QoS requirements for multiple concurrent applications.

Two major QoS requirements, delay and PDR, are considered in this paper. Additionally, multiple flows, each responsible for running an application with its own QoS requirements, are specified. In this paper, we deal with the optimization of several goals at the same time. The QoS optimization problem is identified as a combinatorial optimization problem, and NP-hard [4,5]. Due to the complexity of this multi-objective optimization problem, we developed the QoS-aware Multi-objective Enhanced Differential Evolution Optimization (QMDE) method. This approach facilitates the determination of an optimal TSCH schedule that satisfies QoS requirements, specifically concerning delay and packet loss (critical parameters in industrial environments for each application individually) while concurrently meeting the required throughput for a centralized heterogeneous sensor network.

Achieving the requirements for both delay and packet loss across multiple data flows in WSNs constitutes a multi-objective optimization problem. This is because delay and packet loss are distinct metrics with potentially conflicting goals. Reducing delay might increase the risk of packet loss due to several factors. For instance, in an effort to reduce delay, sensors might transmit data more frequently. This increased volume of data can saturate the network, leading to congestion. Congested networks may drop packets when buffers are full or when packets exceed acceptable transmission deadlines, especially in time-sensitive applications.

In [6,7], the objective was to identify the smallest slotframe size while simultaneously achieving the desired throughput. A minimized slotframe size corresponds to reduced delay [8], although certain applications may tolerate slight delays to prioritize other critical QoS metrics, such as minimizing packet loss.

This study introduces a novel Multi-objective Enhanced Differential Evolution optimization approach aimed at meeting diverse QoS requirements according to the deployed application. Notably, this method represents a pioneering endeavor not previously employed in the existing literature. The main contributions of this work are summarized in the following points:

- We enhanced the Priority-based Customized DE (PCDE) optimization algorithm presented in [7] in terms of time complexity and moved to multi-objective, generating a collision-free and interference-free schedule to address the challenge of meeting QoS requirements across various applications in the industrial wireless sensor network environment.
- We enhanced the PCDE algorithm to schedule a node only when it possesses a packet(s) in its queue to avoid the unnecessary scheduling of transmissions, which is an inefficient use of a TSCH cell.

This paper is structured as follows. We start with a review of related works in Section 2. Following this, Section 3 is dedicated to explaining the problem definition. The methodology of QMDE is detailed in Section 4. Section 5 encompasses the performance evaluation, covering the simulation setup and evaluation results of the proposed approach. The limitations of the algorithm are explained in Section 6. Finally, Section 7 concludes the paper and discusses directions for future research.

## 2. Related Works

The conflict-free TSCH scheduling algorithm, proposed in [9], aims to minimize transmission delays by reducing the slotframe length. The authors introduced the concept of ‘Wave’, where each node transmits data at least once during this period. In this approach, nodes closer to the sink may experience high traffic or queue overflow, leading to increased delays in larger networks. Furthermore, the sensors transmit a fixed number of packets without considering their heterogeneous nature.

Similarly, references [10,11,12,13,14] solely focus on fixed packet rates, disregarding the network’s heterogeneous nature. Additionally, in [15,16], each node is allocated to a single cell, which can degrade algorithm performance under heavy traffic loads.

In the Traffic-Aware Scheduling Algorithm (TASA) [17], matching and coloring principles are employed to determine pairs of transmitting and receiving nodes that do not conflict or interfere. This approach follows a structured approach: initially, it identifies collision-free pairs of transmitter and receiver nodes based on aggregated queue sizes, utilizing matching concepts from graph theory. Subsequently, these selected pairs are assigned to different channels, ensuring interference avoidance through coloring mechanisms. TASA lacks a mechanism to prioritize transmissions based on their required QoS requirements. This means it does not give precedence to specific types of traffic that may need guaranteed levels of service. Furthermore, while TASA performs effectively at very low packet rates, such as one packet per hour, it becomes considerably more complex when handling high packet ratios. This complexity can pose challenges in practical implementations, especially in environments with extensive and varied traffic demands.

The Orchestra scheduling technique, incorporated within the IEEE 802.15.4 standard, provides dynamic and effective coordination for wireless communication in TSCH-based networks [18]. Despite its benefits, Orchestra does face limitations, especially under heavy traffic loads. In these situations, Orchestra scheduling may result in delays and packet loss caused by congestion, ultimately impacting network performance and posing coordination difficulties.

In Deac et al.’s study [19], the goal is to improve Orchestra’s receiver-based scheduling policy for handling high traffic loads. This involves adjusting the static schedule when immediate child nodes connected to the gateway experience congestion. If a child node’s buffer exceeds a set threshold, it notifies the gateway, which then increases reception time slots using a hash function. However, this method may lead to scalability issues as it only tackles collision concerns at the gateway, leaving the problem unresolved in the other nodes throughout the network.

Several studies, including [20,21,22,23], have proposed adaptive strategies for managing data traffic. These approaches suggest methods for dynamically adding or removing cells based on fluctuating traffic conditions.

H. Nguyen-Duy et al. [24] proposed a reinforcement learning (RL)-based scheduling algorithm that utilizes Q-learning for schedule design. Q-learning is a widely used RL technique, particularly effective in small-scale optimization problems. However, in heterogeneous IoT networks, the state space becomes significantly large, rendering tabular methods like Q-learning less efficient [25].

Gyawali et al. [26] applied deep reinforcement learning (DRL) for centralized channel allocation in vehicular networks, but this approach is less effective in dynamic IoT networks where complete channel state information is difficult to obtain. Ye et al. [27] addressed this with a decentralized DRL approach, using actor–critic methods to balance the solution by combining centralized value functions with decentralized policies. Although DQN-based algorithms have been used in industrial environments, they are better suited for real-time and dynamic scenarios, whereas our focus is on optimizing TSCH scheduling in static or semi-static environments.

A range of techniques has been proposed for TSCH scheduling [14,28]. These TSCH scheduling algorithms have been developed with the primary goal of optimizing specific metrics such as delay, energy consumption, or packet delivery rate within the context of a single application, even though industrial wireless sensor networks often support multiple applications running concurrently.

In an industrial environment, each application may have distinct specifications and varying QoS requirements. This scenario results in diverse data traffic, each with its own demands, such as latency and reliability, which must be met to ensure the application’s proper functioning. The challenge lies in devising a TSCH schedule that synchronizes generated packets across diverse applications while meeting their respective QoS criteria. To address these requirements, the focus extends from merely minimizing slotframe size or delay to meet required QoS parameters. In contrast to previous studies that have only focused on minimizing delay [15,19] or maximizing the reliability [11,29,30], this paper deals with the challenge of fulfilling multiple, specified QoS requirements in a heterogeneous wireless sensor network.

Another limitation of current TSCH scheduling algorithms is their underlying assumption of fixed packet rates, which fails to account for the heterogeneity in packet rates that often characterizes real-world applications. Previous research, as outlined in [9,10,11,12,13], often overlooks the impact of heterogeneous packet rates within industrial sensor networks. These studies typically focus on fixed packet rates, which simplifies the estimation of subsequent transmissions for each sensor node. These approaches can lead to be insufficient in scenarios where packet rates vary significantly, as they do not account for the complexities introduced by varying traffic patterns in real-world industrial settings.

Although some solutions have addressed traffic diversity [15,17,19,31], the development of an optimized, differentiated QoS solution for heterogeneous wireless networks remains a significant challenge. To the best of our knowledge, existing approaches do not adequately consider the scenario where multiple applications, each with varying QoS requirements and packet rates, run on a single network. This oversight reveals a substantial gap in the current body of research, as effectively accommodating diverse applications with distinct performance requirements is essential for optimizing industrial sensor networks.

## 3. Problem Definition

Defining data flows is essential to implementing and maintaining QoS policies. QoS parameters such as latency and packet loss requirements can be associated with specific data flows to ensure that they meet the application’s performance objectives. This is critical in industrial environments where reliable and predictable communication is essential for operational efficiency and safety. The following paragraphs elaborate on how these flows are defined and how the applications are assigned to these flows.

A directed tree topology of a wireless sensor network is represented by a graph G=(S,L), where *S* denotes the set of sensor nodes as $S_{1},S_{2},\ldots ,S_{N}$, with *N* representing the total number of sensor nodes, and *L* signifies the set of links. The sensor nodes are arranged in a square grid topology, with the distance between neighboring nodes denoted by $Dist_{NBR}$. It is assumed that all nodes have a uniform communication range, denoted by *R*. This topology is composed of one sink node ($S_{1}$) and $N_{T}$ transmitting nodes ($S_{T}$), each associated with applications having distinct QoS requirements. Each application is defined by a specific packet rate (PR) and is associated with two QoS metrics: the target delay and the acceptable packet loss rate. Moreover, each application is assigned a specific packet rate. The targeted delay and packet loss for application *i* are denoted as $TD_{i}$ and $TL_{i}$, respectively. Since we employ a centralized TSCH scheduling approach, the sink node is assumed to be aware of the target QoS values following an initial information exchange.

**Figure 1:**
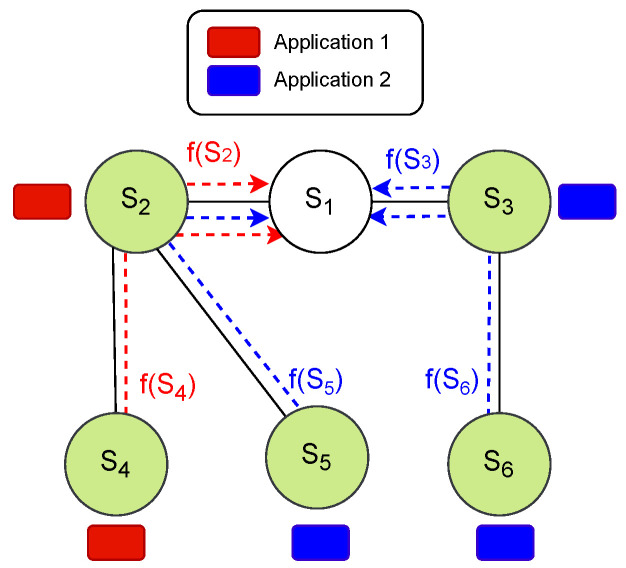
Sample tree topology showing sink, transmitting nodes, and flows.

As applications are allocated to the transmitting nodes, this leads to the creation of multiple flows based on the number of paths from each transmitting node to the sink node. We define a flow as a function $f\left(S_{i}\right):S_{i},\ldots ,S_{1}$, where $S_{i}\in S_{T}$. The primary objective is to ensure that each flow meets its specified QoS requirement. A sample tree topology for six nodes is shown in Figure 1.

As depicted in Figure 1, the sink node is denoted as $S_{1}$, accompanied by five transmitting nodes as $S_{T}=\left\{S_{2},S_{3},S_{4},S_{5},S_{6}\right\}$. In this scenario, two distinct applications, labeled as application 1 and application 2, are introduced. The group of transmitting nodes handling application 1’s packets are identified as $S_{T1}$, and the nodes responsible for transmitting application 2’s packets are labeled as $S_{T2}$. The figure illustrates five flows denoted as $f(S_{2})$, $f(S_{3})$, $f(S_{4})$, $f(S_{5})$, and $f(S_{6})$. Nodes $\left\{S_{2},S_{4}\right\}\in S_{T1}$ are tasked with transmitting application 1’s packets to the sink node $S_{1}$, while sensor nodes $\left\{S_{3},S_{5},S_{6}\right\}$$\in S_{T2}$ are responsible for transmitting application 2’s packets to $S_{1}$. The primary objective is to ensure that each flow satisfies its designated QoS requirements associated with its respective application.

## 4. Methodology

**Figure 2:**
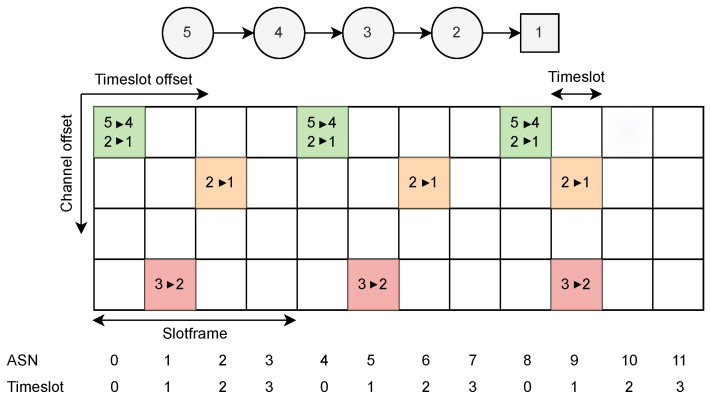
Simple wireless network topology with an example TSCH schedule.

A TSCH schedule is structured in the form of a matrix, as illustrated in Figure 2, encompassing channel offsets and time slot offsets to represent individual cells, with groups of time slots forming a slotframe. Here, the slotframe consists of 4 time slots, and there are 4 channel offsets. A cell is indicated by a tuple (timeslotOffset, channelOffset), and it can be shared by multiple transmissions or dedicated to a single transmission. One time slot provides enough duration for the transmitter to send a maximum-length packet and for the receiver to transmit an acknowledgment in response. The network coordinator is responsible for the management and control of traffic flows, and it computes the optimized time slot and channel assignment based on a particular objective.

The coordinator node periodically broadcasts Enhanced Beacons (EBs), which contain the current Absolute Slot Number (ASN). The ASN is the total number of time slots elapsed since the deployment of the network, as illustrated in Figure 2.

In the process of developing the QMDE, we were driven by the following objectives:

- Attain the specified throughput requirements considering the packet rate for the nodes.
- Fulfill the predetermined latency criteria, ensuring that the delay for all nodes remains within the defined limit.
- Achieve a packet loss rate that meets or falls below the specified QoS threshold to improve the overall reliability of the network.

**Figure 3:**
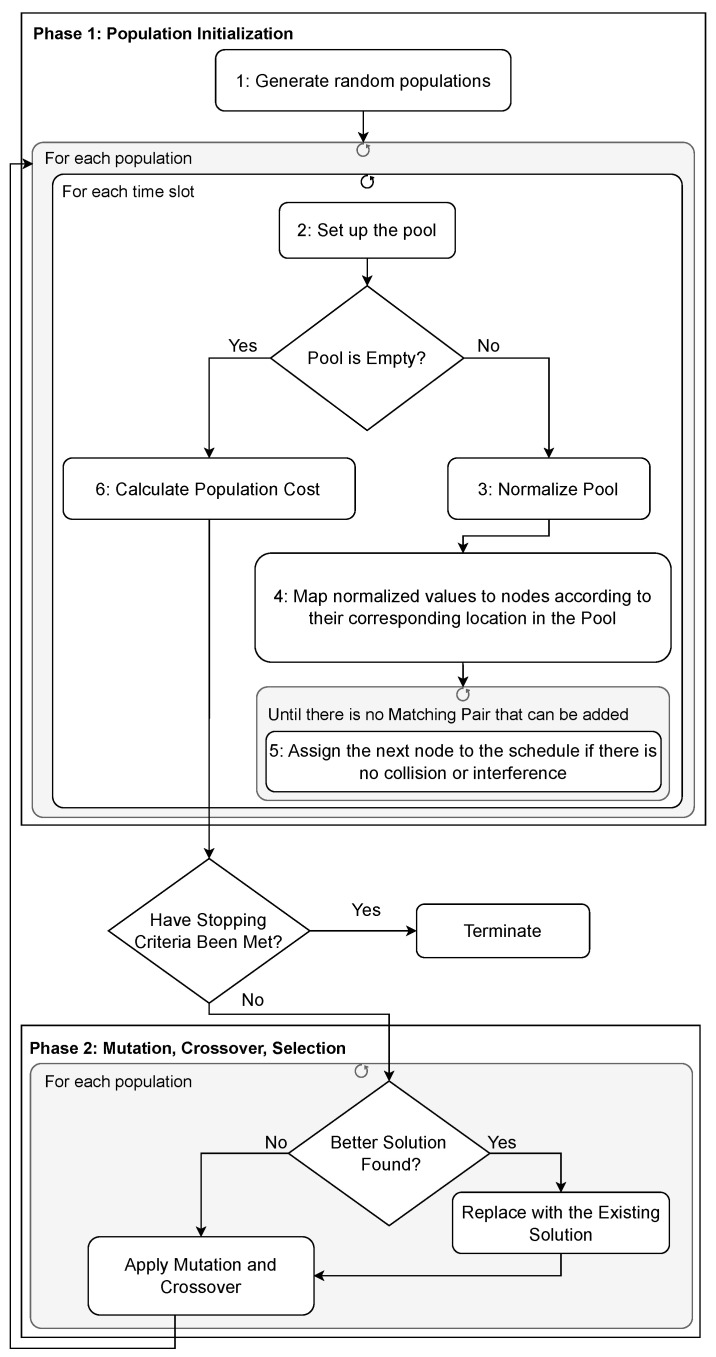
QoS-oriented Multi-objective Differential Evolution Optimization flowchart.

Figure 3 illustrates the flowchart detailing the general process of QMDE. To clarify this process, we have divided it into two phases. Phase 1 focuses on population initialization, while phase 2 is dedicated to the mutation, crossover, and selection stages of the optimization process. In the following, each phase is explained in detail.

### 4.1. Phase 1: Population Initialization

In this phase, we explain how the initial population for optimization is created and transformed into a TSCH schedule through various steps. Furthermore, we elaborate on the module responsible for identifying sensor nodes that can be added to a time slot without causing collisions or interference, along with how we calculate the fitness value for the resulting schedule. The following paragraphs outline the steps depicted in Figure 3.

Step 1: Initially, a set of $n_{Pop}$ random vectors (initial population) is generated. Each population is represented as $Pop_{i}$, a vector of size m×n, where *m* denotes the number of channels and *n* signifies the maximum slotframe size. $Pop_{i}$ consists of values ranging between $Var_{min}$ and $Var_{max}$. Here, $Var_{min}$ is assumed to be 1, while the $Var_{max}$ is determined as the total expected number of transmissions, calculated using the following equation:

(1) $$Var_{max}=\sum_{i=2}^{N}\left\lceil L_{sf}\cdot PR\left(S_{i}\right)\cdot D\left(S_{i}\right)\right\rceil ,$$

where $PR(S_{i})$ represents the packet rate of sensor node $S_{i}$ and $D(S_{i})$ denotes the depth of the sensor node $S_{i}$ in the tree structure. Moreover, $L_{sf}$ is the slotframe length, which is calculated by Equation (2):

(2) $$L_{sf}=L_{ts}\cdot N_{ts},$$

where $L_{ts}$ is the time slot length, which is assumed to be a standard value of 10 ms, and $N_{ts}$ represents the number of time slots in a slotframe.

Finally, in this step, each $Pop_{i}$ is transformed into an m×n matrix to align with the dimensions of a candidate TSCH schedule. The values in this matrix are used to map out transmissions, ultimately forming a candidate TSCH schedule by the end of step 5.

Step 2: The TSCH schedule is built based on a pool, which contains sensor nodes with packets ready for transmission. The pool is established separately for each time slot. We introduced the pool concept to store eligible sensor nodes in each time slot that is available for scheduling. Eligible nodes are those with packets in their queues, either their own or destined for relaying to the sink node. Utilizing this pool concept, the QMDE algorithm ensures that nodes responsible for relaying packets are not scheduled until they receive at least one packet from their children or generate one themselves. It is crucial to monitor which sensors have packets ready for transmission to avoid scheduling sensors without packets, leading to unnecessary listening at scheduled times and resulting in energy inefficiency.

**Figure 4:**
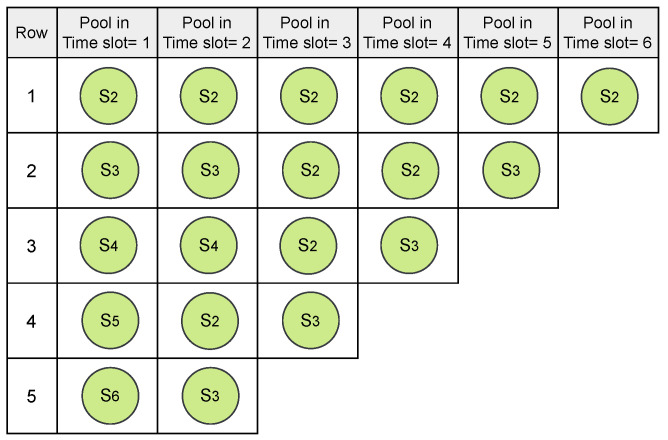
Sample of six pool statuses corresponding to six time slots.

In the example illustrated in Figure 1, and assuming that all packets are generated in network initialization, during the first time slot with an empty pool, $S_{2}$, $S_{3}$, $S_{4}$, $S_{5}$, and $S_{6}$ are eligible for addition to the pool in the first time slot. Consequently, these five nodes form the pool in time slot 1, as shown in Figure 4.

**Figure 5:**
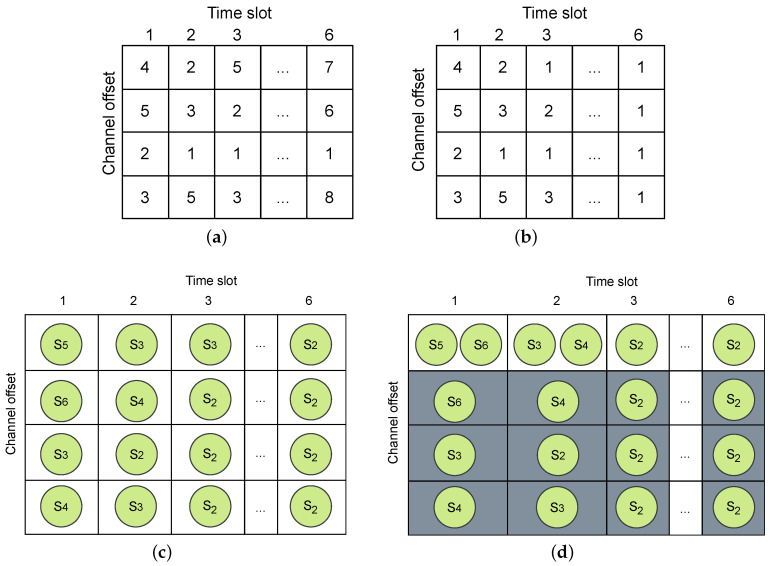
Process of mapping the generated matrix values to sensors for TSCH schedule creation: (**a**) random matrix generation, (**b**) normalization, (**c**) mapping the sensor’s position in the pool, and (**d**) assign nodes and matching pairs.

Starting with an empty pool in each time slot, if a sensor node is assigned to the TSCH schedule in the previous time slot and has no remaining packets in its queue, it will not be added to the pool in the next time slot. Conversely, if the sensor node is not assigned yet, it will be moved to the pool in the next time slot until it has no remaining packets in its queue. As depicted in Figure 5d, $S_{5}$ and $S_{6}$ are allocated to time slot 1. Consequently, they are not included in the pool in time slot 2, as demonstrated in Figure 4. Additionally, nodes $S_{2}$ and $S_{3}$, now with two expected packets, will be added to the pool in time slot 2. These two nodes are now responsible for relaying received packets from their child nodes toward the sink node, in addition to transmitting their own generated packets. Then, in time slot 2, $S_{3}$ and $S_{4}$ are assigned (Figure 5d), resulting in the pool being updated. This process continues until no more transmissions are left to schedule. A sample of six pool statuses for six time slots is shown in Figure 4.

The PoolSize for each time slot corresponds to the total number of expected packets that can be transmitted in a time slot.

Step 3: During this step, the values in the column of the matrix $Pop_{i}$, which corresponds to the specific time slot in the TSCH schedule, are normalized based on the obtained PoolSize for that particular time slot. During the normalization process, if a cell value in the matrix exceeds the pool size value, it is replaced by the cell’s value module of the PoolSize. Normalization ensures that the values in each time slot of the $Pop_{i}$ matrix fall within the same range as the associated pool size.

To better explain the normalization process, we provided an example. In Figure 5a, a 4 × 6 matrix has been generated randomly in step 1. The values in this matrix, ranging from 1 to 8, represent the total number of expected packets to be transmitted in the network topology depicted in Figure 1. In Figure 5a, values in time slot 1 should be normalized to 5 (PoolSize in time slot 1, as depicted in Figure 4). Similarly, values in time slot 2 should also be normalized to 5. Since the values in these two time slots are already equal to or less than the corresponding pool size, they remain unchanged. However, in time slot 3, the expected number of transmissions is four rather than five, as shown in Figure 4 (PoolSize=4). After normalization, the values in time slot 3 of the matrix depicted in Figure 5a as 5, 2, 1, and 3 are updated to 1, 2, 1, and 3 as demonstrated in Figure 5b. Any value equal to or greater than 4 is normalized. That is why 5 is normalized to 1 (5 mod 4), and the rest of the values are unchanged. The process of normalization continues for time slots 4, 5, and 6, where the matrix values are normalized based on the pool size in each corresponding time slot. The values shown in Figure 5a are normalized, as depicted in Figure 5b.

Step 4: Following normalization, step 4 involves assigning sensor nodes to the TSCH schedule. This assignment is accomplished by mapping the normalized matrix values in each time slot from Figure 5b to sensor nodes located in the corresponding pool based on their respective row positions in the pool. As illustrated in Figure 5b, the values in time slot 1 shown as 4, 5, 2, and 3 are mapped to $S_{5}$, $S_{6}$, $S_{3}$, and $S_{4}$, as demonstrated in Figure 5c, and corresponding to the row numbers in the pool depicted in Figure 4 for time slot 1. This mapping process is repeated for each time slot.

Step 5: In this step, each node assigned to a specific cell attempts to include all its Matching Pairs (MPs) from the pool into that particular cell. Matching pairs refer to nodes already in the pool that do not cause any collision or interference with the transmissions that are already scheduled. The addition of matching pairs continues until there are no remaining matching pairs available that can be added to the time slot ts.

The operations of updating the pool and its size value, normalization, mapping, matching pair addition, and node assignment are iteratively performed for each time slot until the pool becomes empty, indicating all necessary transmissions have been scheduled.

Step 6: After constructing the schedule for each population, the cost for each schedule is determined. We calculate the cost for the generated schedule in terms of delay and packet loss. The QMDE algorithm generates essential configuration files that are distributed to the nodes in the network. In our case, we leverage a network simulator, although an actual sensor network could be used. The simulator evaluates the provided schedule based on various quality of service metrics. After the simulator runs, the results show values for delay, throughput, and packet loss, providing insights into the network’s QoS performance with the generated schedule. The outcome of this step is a vector that contains the delay and packet loss of each flow $f(S_{i})$.

If any of these populations meet the QoS requirements for the applications, the QMDE algorithm terminates. Otherwise, it proceeds to phase 2 of the QMDE algorithm.

### 4.2. Phase 2: Mutation, Crossover, and Selection

In phase 2, our focus shifted to optimizing the populated generation using an improved version of the customized differential evolution (CDE) algorithm. We initially introduced the CDE algorithm in [32]. However, in QMDE, we have refined it further, particularly in the population initialization and selection steps. These adjustments aim to generate better schedules and select a population capable of achieving QoS objectives for the defined applications in QMDE.

The CDE algorithm involves adapting the traditional differential evolution (DE) algorithm [33] to effectively manage the challenges posed by the inconsistent number of variables and variable sizes inherent in TSCH scheduling. This adaptation was crucial due to TSCH’s capability to support concurrent transmissions within a single time slot or cell. CDE generates TSCH schedules by applying crossover and mutation operations and mapping values to collision-free and interference-free pairs of nodes.

In QMDE, we not only assign collision-free and interference-free transmissions to specific cells or time slots but also employ a pool concept. This method facilitates the generation of schedules based on packets that are prepared for scheduling to avoid over-scheduling and energy inefficiency. Moreover, the selection process is enhanced to support multi-objective optimization, ensuring that various QoS metrics are met effectively.

In phase 1, we generated a set of $n_{Pop}$ populations, denoted as {$Pop_{1},Pop_{2},\ldots ,Pop_{n_{\mathrm{pop}}}$}. As shown in Figure 3, during each iteration, if the calculated fitness value of the multi-objective function for the generated population $Pop_{i}$ indicates that the population fails to meet the requirements, mutation operation is performed to generate a candidate population, referred to as $V_{i}$ as shown below:

(3) $$V_{i}=Pop_{R_{1}}+F\times \left(Pop_{R_{2}}-Pop_{R_{3}}\right),$$

where $R_{1}$, $R_{2}$, and $R_{3}$ are non-equivalent random numbers that are used to choose up to three random populations from the total population and then generate a candidate population as $V_{i}$ from these populations. The function *F* is a mutation factor.

For each population $Pop_{i}$, QMDE creates a new $V_{i}$ population. The mutation step is responsible for generating new candidate solutions by introducing small modifications to existing solutions.

Then, by using a crossover operation, the algorithm blends components from both the original and mutated population $V_{i}$ to generate the new population as $NewPop_{i}$. We used binomial crossover, where components from the candidate population $V_{i}$ replace corresponding components from $Pop_{i}$ with a certain probability $P_{CR}$ (crossover rate).

(4) $$NewPop_{i}=\left\{\begin{array}{cc} V_{i} & \mathrm{if}\;rand_{j}\leq P_{CR}\;\mathrm{or}\;j=i\\ Pop_{i} & \mathrm{otherwise} \end{array}\right.$$

where $rand_{j}$ is a random number for each component *j*.

Following crossover, the generated population is subjected to pool setup, normalization, mapping, and MP pair addition procedures (steps 2 to 5) in order to generate a candidate solution $NewPop_{i}$. If the new candidate solution $NewPop_{i}$ proves to be better compared to the current solution $Pop_{i}$, it replaces the existing solution. The better solution refers to a solution with smaller values for $F_{D}$ and $F_{L}$, as shown in Equation (5). Otherwise, if the newly generated candidate is not better, the optimization continues until one of the following stopping criteria is met: (1) the number of iterations exceeds the defined maximum value, MaxIter, or (2) the populated schedule fulfills the primary objectives set by applications.

The multi-objective optimization problem used in the QMDE algorithm is shown in Equation (5). The scope of this optimization problem can be expanded to accommodate additional applications within the network with more QoS requirements. Since we considered two QoS requirements in this paper, there are consequently two objectives ($F_{D}$ and $F_{L}$), as outlined in Equation (5).

(5) $$\begin{array}{cc} \; & F_{D}=\frac{\sum_{i\in S_{T1}}f\left(S_{i}\right).d-TD_{1}}{size(S_{T1})}+\frac{\sum_{i\in S_{T2}}f\left(S_{i}\right).d-TD_{2}}{size(S_{T2})},\\ \; & F_{L}=\frac{\sum_{i\in S_{T1}}f\left(S_{i}\right).ls-TL_{1}}{size(S_{T1})}+\frac{\sum_{i\in S_{T2}}f\left(S_{i}\right).ls-TL_{2}}{size(S_{T2})},\\ \; & Obj=Minimize\{F_{D},F_{L}\}. \end{array}$$

The first objective, denoted as $F_{D}$, aims to meet the delay requirements for nodes executing application 1 ($S_{T1}$) and application 2 ($S_{T2}$). The maximum target delay for these two applications is denoted as $TD_{1}$ and $TD_{2}$, respectively. The variables $f(S_{i}).d$ represent the end-to-end delay of node $S_{i}$. $F_{D}$ calculates the average difference between the transmitting nodes’ delay in the candidate schedule and the required delay for each associated application.

Similarly, $F_{L}$ is formed to meet the packet loss requirements for two defined applications, following the same approach of evaluating the average difference between the packet loss of nodes running applications 1 and 2 in the candidate schedule and the targeted packet loss for associated applications. Target packet loss for applications 1 and 2 are specified as $TL_{1}$ and $TL_{2}$, respectively. The packet loss of sensor $S_{i}$ in the candidate schedule is represented by $f(S_{i}).ls$. The main goal is to minimize the values of $F_{D}$ and $F_{L}$, aiming to reduce the difference between the target and actual delay and packet loss for each flow.

To enhance the optimization process’s effectiveness and capture a broader range of solutions, we define a candidate optimal solution as one where either the delay or the PDR improves, even if the other metric deteriorates. Specifically, we consider a solution as a candidate if, for instance, the PDR improves while the delay worsens, or vice versa, provided that the deterioration does not exceed predefined thresholds. These thresholds are set at 5 milliseconds for delay and 15% for PDR.

## 5. Performance Evaluation

This section outlines the simulation setup and provides a comprehensive evaluation of the QMDE approach. We explore the impact of different factors on network performance, particularly focusing on variations in the number of nodes and traffic rates. By adjusting the percentage of nodes allocated to specific applications, we modulate the network traffic rate to analyze its influence on overall performance.

The implementation of QMDE was achieved through co-simulation using Matlab and TSCH-SIM to obtain more accurate results. We also enhanced the TSCH-SIM simulator [34] by incorporating manual routing and scheduling features in [35]. The ability to manually configure the TSCH schedule and static routing within the TSCH-SIM simulator allows for testing the TSCH schedule across various configurations. Further details about the implementation of manual routing and scheduling in TSCH-SIM are provided in [35].

**Figure 6:**
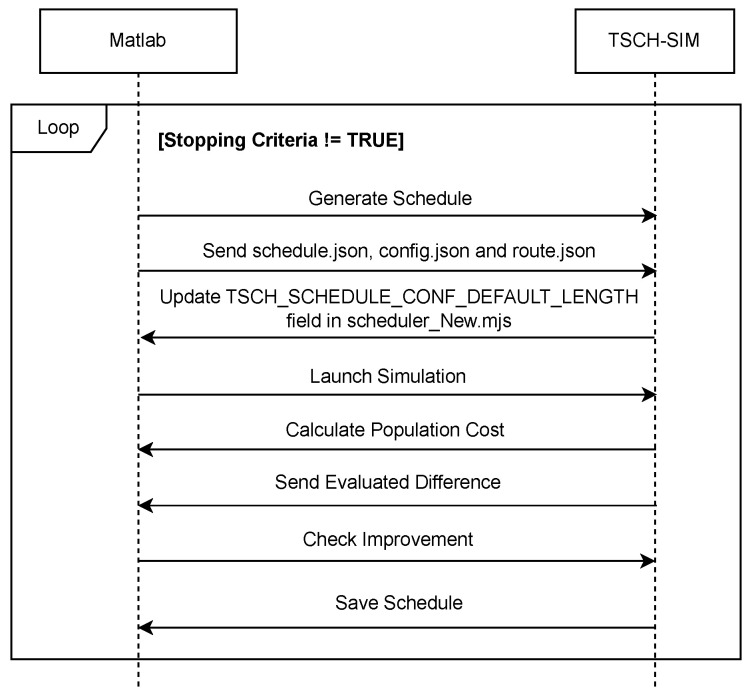
Co-simulation: sequence diagram of QMDE using Matlab and TSCH-SIM.

We employed Matlab for the optimization process and TSCH-SIM for network simulation to determine QoS values. The entire process of refining the TSCH schedule to achieve the optimal solution is conducted offline. This approach ensures that the network operations are not halted during the optimization process; instead, the network continues to function uninterrupted while the optimal schedule is being determined. Figure 6 illustrates the detailed sequence diagram for this co-simulation. Further details regarding the co-simulation between TSCH-SIM and Matlab can be found in [36].

Initially, nodes are randomly distributed within a 2D environment. Subsequently, employing the MST, sensors with shorter distances between them are linked through a tree topology, resulting in the creation of an interconnected sensor network. This tree is utilized to establish the pathway from any sensor node to the sink node. Each node is equipped with information regarding its location and packet rate, with the sink node being the sole entity possessing packet rate and spatial information for all nodes. This information is crucial for the sink node to generate a schedule, distribute it to all nodes, and enable efficient switching between listening and sleeping modes for packet transmission or energy conservation based on the schedule. Subsequently, applications are assigned to the transmitting nodes, each with its own set of QoS requirements.

### 5.1. Simulation Setup

To evaluate the proposed algorithm, we carried out a co-simulation between Matlab and TSCH-SIM [36]. The simulation configuration consists of deploying 16 to 100 nodes with a constant communication range of R=30 m distributed in a squared grid topology. The list of parameters and their ranges are defined in Table 1. The sink node is located at the center of the grid, and the Minimum Spanning Tree (MST) algorithm was employed to generate a tree topology for the sensor network.

As outlined in the previous section, transmitting nodes are associated with an application class. Each application has a defined packet rate and required QoS. The specified QoS requirements for each application align with the guidelines outlined in the ISA SP100 Standard [37,38,39]. This standard classifies industrial applications into six categories, with our focus primarily on class 2: closed-loop supervisory control, and class 4: condition monitoring.

In a class 2 application, such as a PLC-based supervisory system, PLCs (Programmable Logic Controllers) send commands to actuators to carry out tasks. In discrete manufacturing, tasks are often completed in a sequence. Therefore, any delay in the communication link between the supervisor (PLC) and the actuators can directly affect the production speed. This means that higher latency in these communications can slow down the overall production process. However, applications associated with class 4 are less critical in terms of latency. Typical applications of class 4 consist of sensing and monitoring devices such as event-based maintenance applications.

Application 1 mimics a closed-loop control system (class 2), which is often critical in terms of delay. Application 2 mimics a condition monitoring application of class 4. Application 1 necessitates a delay of less than 50 ms and a packet loss of under $10^{-7}$. Application 2 functions as a condition monitoring application, with a latency requirement of less than 100 ms and a target packet loss rate of under $10^{-6}$. In this type of application, the system is utilized for data collection and forwarding to a server. For example, nodes are deployed to gather data, such as temperature, over a specific period. These collected data can then be used to inform long-term temperature management strategies. The specifications for each application are clearly outlined in Table 2.

We have categorized packet rates into the following two groups and assigned a specific packet rate group to each node based on the application it is running. Sensor nodes running application 1 are allocated to the medium-rate category (M), while sensor nodes executing application 2 will be assigned to the low-rate (L) category, as shown in Table 2.

Table 3 presents five primary scenarios used to assess the performance of the QMDE algorithm. Each scenario is evaluated across a range of sensor counts, varying from 16 to 100. This results in four sub-categories for each scenario. Thus, our approach is tested across a total of 20 unique scenarios.

In the optimization part of the QMDE algorithm, we implemented the initial optimization parameters outlined in Table 1. MaxIter represents the maximum number of iterations, while $n_{Pop}$ denotes the initial number of populations. The size of the differential evolution decision variable is equal to the maximum size of the TSCH schedule (m×n), where *m* is set to a fixed value of four channels, and *n* is the slotframe size and set to 500. It is important to emphasize that the selection of 500 time slots as the maximum slotframe size does not mandate the use of all time slots; rather, it offers a range of random numbers for potential selections. For instance, if the pool is emptied by time slot 10, the remaining 490 time slots are not utilized and are deleted.

$Var_{min}$ is set at 1 while $Var_{max}$ represents the total number of transmissions determined by Equation (1). In Table 1, the Crossover Probability ($P_{CR}$) determines the likelihood of using the crossover operation to generate the new population by recombining elements from the current population. A higher $P_{CR}$ emphasizes exploration, while a lower value prioritizes exploitation. We used 0.8 since we wanted to increase the probability of generating new solutions. The scaling factor, defined by $beta_{min}$ and $beta_{max}$, controls the adjustment of difference vectors during mutation. A smaller scaling factor intensifies local exploration, while a larger factor broadens the search space.

Table 4 illustrates the simulation parameters and corresponding values that were taken into account for the simulation.

### 5.2. Simulation Results

In this section, we discuss the outcomes of our experiments on QMDE, which were conducted using a co-simulation between Matlab and TSCH-SIM. We evaluated several key metrics, including delay, reliability, slotframe size, and time complexity. Additionally, we compared these results with those obtained from the TASA algorithm, focusing specifically on delay and Packet Delivery Ratio (PDR). This comparison helps us understand how QMDE performs relative to TASA in different scenarios.

#### 5.2.1. Experiment 1: Achieving the QoS Values

Network delay refers to the total time a packet takes to travel from a source node to a destination node. Here, the delay is evaluated by taking the difference between the time a packet is generated and is successfully received by the root node. On the other hand, reliability denotes the network’s capability to transmit data successfully between the sender and receiver, often quantified using an end-to-end PDR.

**Figure 7:**
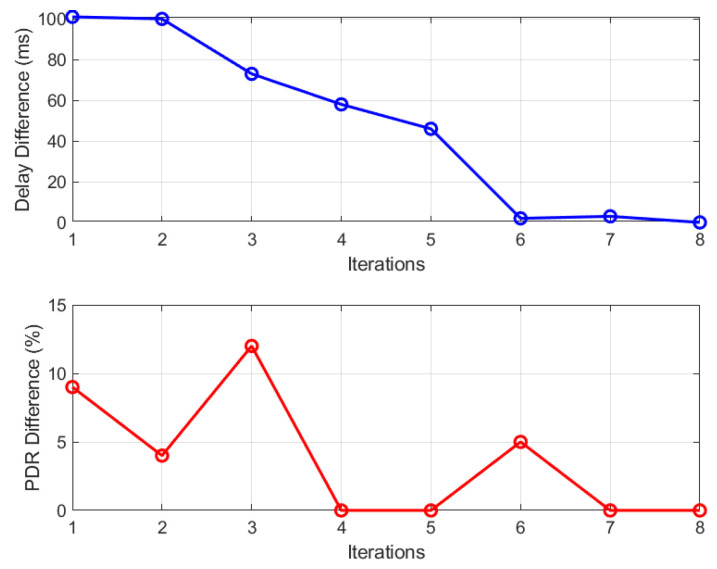
Optimization progress in scenario 5 with 64 nodes.

Figure 7 illustrates the delay and PDR differences for candidate solutions in scenario 5 with 64 nodes. In each optimization iteration, a solution is considered a candidate if either the delay or PDR difference improves, even if the other metric worsens slightly but remains within acceptable limits. This explains the fluctuations seen in Figure 7. If neither metric shows improvement, the optimization process continues until it meets the termination criteria.

In Figure 7, iteration 1 begins with a schedule showing a delay difference of nearly 100 ms and a PDR difference of 9%. This schedule is replaced with a better one in iteration 2, featuring a reduced PDR difference (4%) and a nearly unchanged delay difference. As discussed earlier, a solution qualifies as a candidate if, for example, the PDR improves while the delay worsens, or vice versa, provided the degradation stays within predefined thresholds. These thresholds are set at 5 ms for delay and 15% for PDR.

By iteration 3, the delay difference reduces by 25 ms, but the PDR difference increases by 14%. In iteration 3, despite the increase in PDR difference falling below the defined threshold, the discovered schedule qualifies as a candidate solution due to its improved delay difference. Ultimately, as depicted in Figure 7, the QMDE algorithm successfully identifies the optimal solution by iteration 8, resulting in zero difference between the defined QoS targets for two applications and the achieved QoS values from the optimal solution.

This behavior is similar for all other scenarios except for scenarios involving 100 or more sensor nodes. For those scenarios, it was not possible to achieve the specified delays for the applications. Given that the time slot duration is 10 ms, the maximum allowed delay for nodes running application 1 corresponds to 5 time slots and for nodes running application 2 it is 10 time slots. Consequently, finding a transmission arrangement that meets these constraints is not possible.

#### 5.2.2. Experiment 2: Slotframe Size

**Figure 8:**
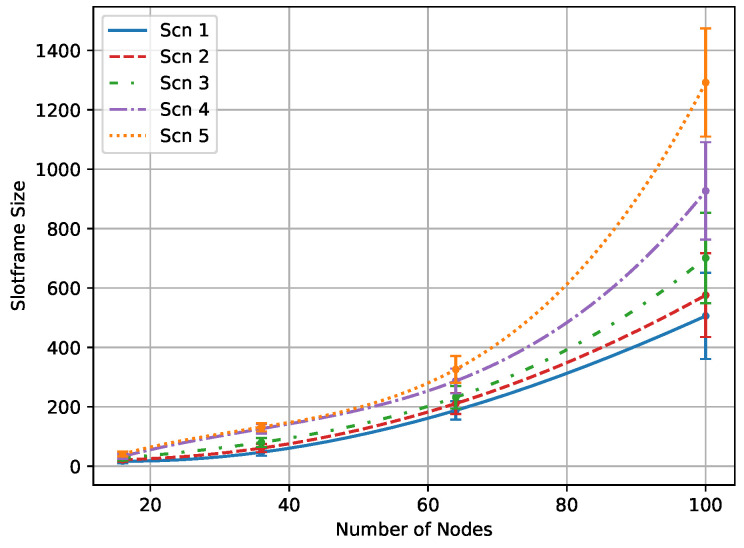
Slotframe size of QMDE algorithm in various scenarios.

The size of the slotframe significantly impacts delay and is determined by the total number of time slots within the slotframe. According to Figure 8, it can be inferred that achieving the defined QoS for the application is influenced by two main factors: increasing the number of nodes and increasing the percentage of nodes with higher packet rates. As seen in the scenarios, higher percentages of nodes with higher packet rates exponentially increase the number of required time slots to meet PDR or delay goals. If the optimization process does not find the optimal schedule, it increases the slotframe size until the termination criteria are met.

#### 5.2.3. Experiment 3: Delay

**Figure 9:**
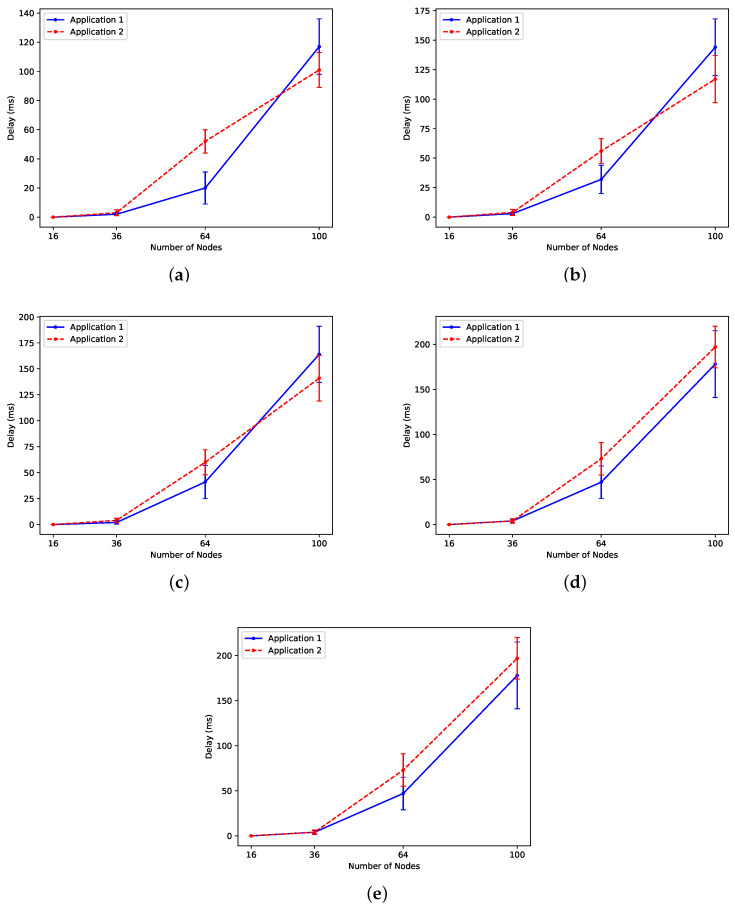
Evaluation of delay between applications in (**a**) Scn 1, (**b**) Scn 2, (**c**) Scn 3, (**d**) Scn 4, and (**e**) Scn 5.

The delay for each flow is defined as the time difference between when the packet is transmitted and when it is received by the sink node. As mentioned earlier, the expected delay for sensors executing application 1 should not exceed 50 ms, while sensors executing application 2 should maintain a maximum delay of 100 ms. The QMDE algorithm successfully fulfills these pre-determined delay criteria for both applications across scenarios involving 16, 36, and 64 sensor nodes, as illustrated in Figure 9. However, for the network size of 100 nodes, the obtained delay values are a little higher than the requirement for the considered scenarios. Given that the time slot duration is 10ms, the maximum allowed delay corresponds to 5 time slots for nodes running application 1 and 10 time slots for nodes running application 2. Consequently, finding a transmission arrangement that meets these constraints becomes considerably impossible.

We observed that the type of application running on nodes near the sink node plays a crucial role. Each application has a specified packet rate, and when nodes close to the sink execute an application with a higher packet rate, this leads to higher delays. Managing traffic and achieving the specified delay becomes challenging because these nodes are tasked with transmitting their packets while bearing the traffic burden from other nodes.

#### 5.2.4. Experiment 4: Reliability

**Figure 10:**
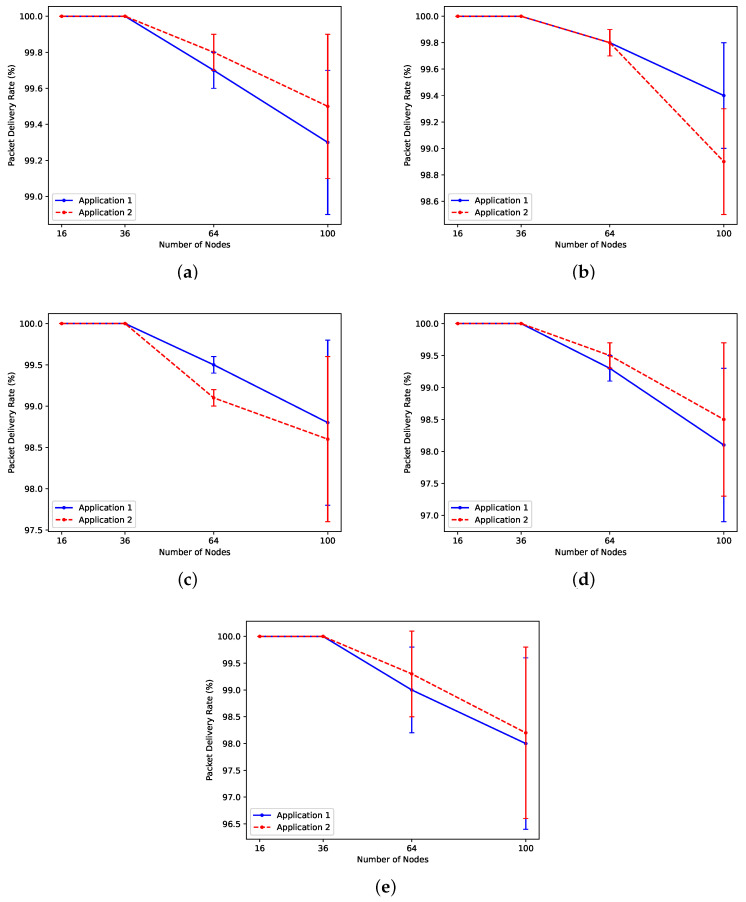
Evaluation of PDR for two applications in (**a**) Scn 1, (**b**) Scn 2, (**c**) Scn 3, (**d**) Scn 4, and (**e**) Scn 5.

The QMDE algorithm showcases its efficacy in identifying an optimal schedule that meets the specified PDR. While in certain scenarios, the algorithm may require additional time to identify the schedule, it can reliably find a schedule, as depicted in Figure 10. In scenarios featuring 16 and 36 nodes, the optimal schedule achieves a perfect PDR of 100%. However, as the number of nodes increases to 64 or more, this PDR value decreases slightly. The algorithm explores different candidate solutions, some of which prioritize minimizing delay while others prioritize maximizing PDR. Balancing these opposing objectives becomes challenging in certain scenarios. Nevertheless, the optimization process continues until it identifies the optimal schedule, striking a balance between PDR and delay.

#### 5.2.5. Experiment 5: Time Complexity

**Figure 11:**
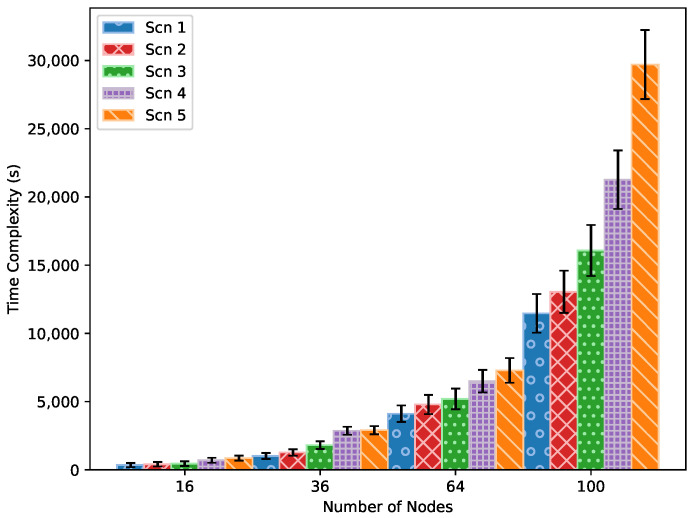
Time complexity of QMDE algorithm in various scenarios.

As shown in Figure 11, increasing the number of nodes and the percentage of transmissions with higher packet rates requires more iterations and time for the QMDE algorithm to find the optimal schedule. In scenarios with 16 to 36 nodes, the probability of packet loss due to queue overflow or high delay is minimal. This is because of the lower number of required transmissions in these scenarios, leading to a smaller slotframe size. As a result, the generated schedule should be capable of meeting the QoS requirement, potentially eliminating the need for numerous iterations to find the optimal schedule. As the number of nodes increases, finding the optimal schedule becomes more challenging. Due to the lack of precise information about when nodes generate packets, it becomes time-consuming to assign transmissions to appropriate time slots without exceeding the expected delay or reducing the desired PDR. This challenge persists for scenarios with a higher percentage of nodes transmitting at higher packet rates.

Figure 11 illustrates that the time complexity of the QMDE optimization algorithm is exponential with respect to the number of nodes. Nevertheless, due to its offline nature, there is no requirement to interrupt the network or repeatedly apply the algorithm. Once the optimal TSCH schedule is determined, it is distributed among nodes, ensuring efficient operation without continuous adjustments.

While it is important to consider the overhead associated with the proposed QMDE algorithm, it should be clarified that this process is performed only once and requires recalculation only if the network topology changes, which is infrequent. The computational complexity of the QMDE algorithm involves three main components: population initialization, mutation and crossover operations, and fitness evaluation. Population initialization requires setting up $n_{Pop}$ individuals, each represented by a matrix with *D* dimensions, where D=m×n (with *m* as the maximum slotframe size and *n* as the number of channel offsets). This step involves $O(n_{Pop}\cdot D)$ operations. Mutation and crossover operations are applied to each individual in the population, each requiring $O(n_{Pop}\cdot D)$ operations per generation. Fitness evaluation in TSCH-SIM depends on the average packet rate of sensor nodes $Avg_{PR}$, the total number of nodes (*N*), and the simulation time ($T_{s}$). Assuming the fitness function evaluation has a complexity of O(Simulator) per iteration, the combined computational complexity of these components is $O(n_{Pop}\cdot D\cdot Simulator\cdot MaxIter)$.

#### 5.2.6. Experiment 6: Delay Comparison between QMDE and TASA

**Figure 12:**
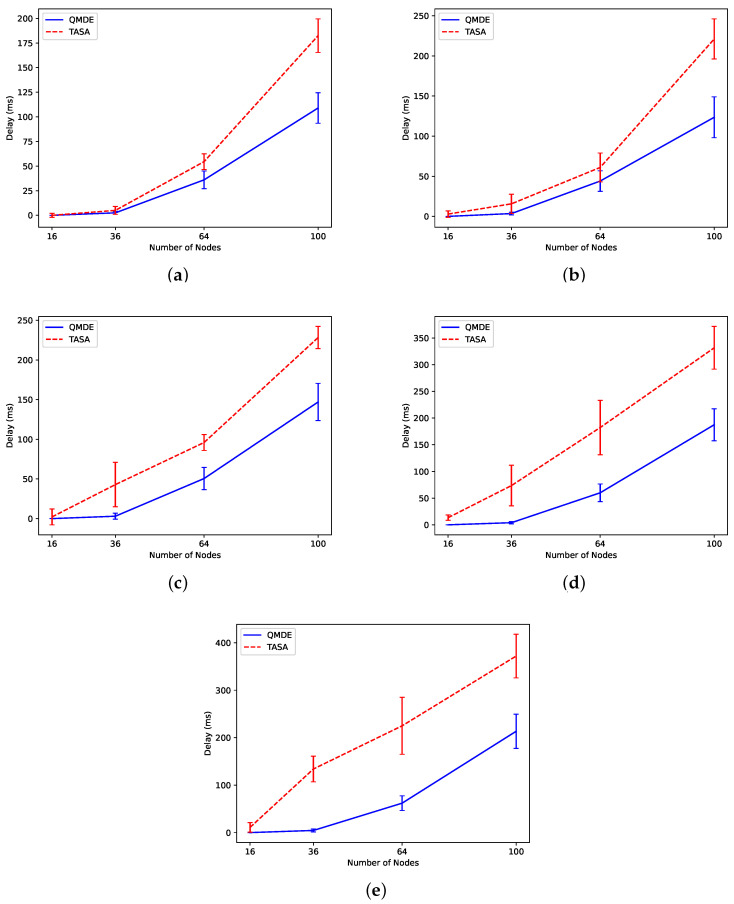
Delay comparison between QMDE and TASA in (**a**) Scn 1, (**b**) Scn 2, (**c**) Scn 3, (**d**) Scn 4, and (**e**) Scn 5.

Regarding the delay, Figure 12 illustrates the average delay observed when two applications (application 1 and application 2) run simultaneously using QMDE and TASA approaches. The average delay provides an overview of the overall delay performance across all defined flows in the network. The figure clearly demonstrates that TASA consistently illustrates higher delay values compared to QMDE. Additionally, as the number of nodes increases, and particularly when a larger proportion of nodes execute application 1 (which has a higher packet rate than application 2), the average delay increases significantly in TASA.

TASA assumes that all packets have been generated in the network initialization step and creates the schedule by prioritizing the nodes with a higher number of packets in their queue, leading to an unbalanced schedule for the case when two applications with different QoS requirements are running. Since there is no access to the exact packet generation time in real networks, nodes may be scheduled before they have packets ready, resulting in an increased delay as they wait until the next scheduled transmission slot. The QMDE algorithm optimizes the TSCH schedule by dynamically adjusting the assignment of transmissions through updating the pool, crossover, and mutation, thereby reducing delay. By individually optimizing the schedule for each application, QMDE identifies the best time slots for each transmission to meet the target delay for each flow effectively.

#### 5.2.7. Experiment 7: Packet Delivery Ratio Comparison between QMDE and TASA

**Figure 13:**
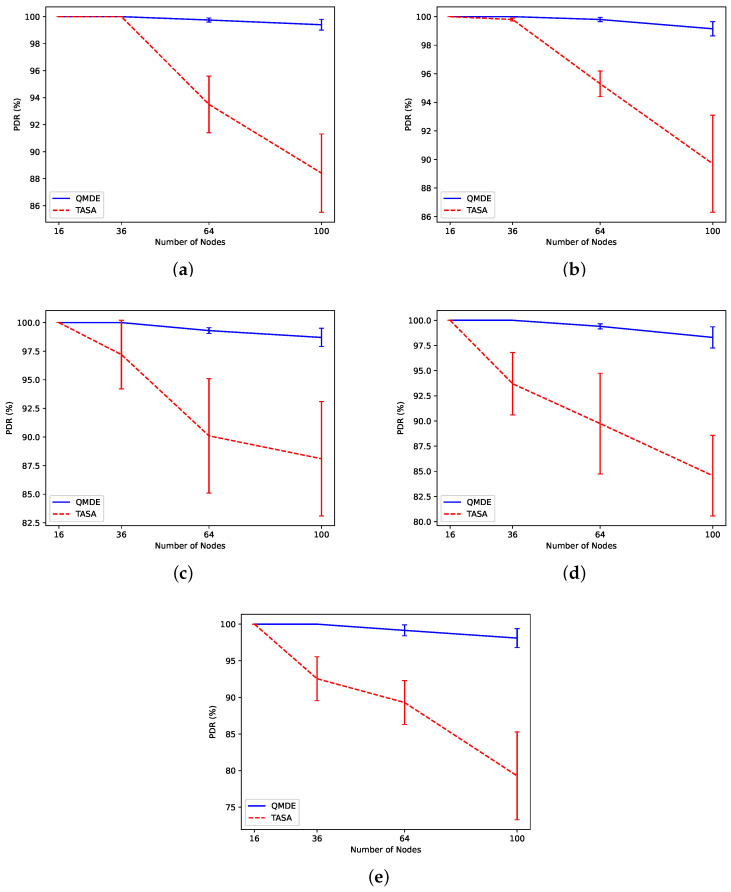
PDR comparison between QMDE and TASA in (**a**) Scn 1, (**b**) Scn 2, (**c**) Scn 3, (**d**) Scn 4, and (**e**) Scn 5.

As depicted in Figure 13, QMDE maintains a high PDR, despite experiencing a slight decrease in scenarios with a higher number of nodes. In contrast, TASA prioritizes scheduling nodes with a greater number of packets first, leading to potential packet loss for nodes experiencing buffer overflow or long delays. Although TASA reaches a high PDR for a lower number of nodes (16 and 36 nodes), this value decreases by increasing the number of nodes and increasing the network traffic. QMDE optimizes the scheduling process by iteratively adjusting the schedule, ensuring that each application achieves the required PDR. This optimization involves strategically assigning sensor nodes to appropriate time slots and ensuring that the necessary number of nodes are allocated to meet the target PDR.

## 6. Discussion

While extensive experiments and analyses were conducted using the network simulator to evaluate the performance of the algorithm in various scenarios, there are limitations compared to real-world network conditions. For instance, the distribution of configuration information in simulators such as TSCH-SIM is often simplified; schedules are usually specified in a single file distributed to all nodes. However, in real-world deployments, this process is complex and managed by the IEEE 802.15.4e beacons. Such simplifications can lead to discrepancies between the simulated and actual performance.

As with every centralized algorithm, the QMDE algorithm requires the coordinator node to have complete topology information and knowledge of sensor nodes’ traffic rate. The network coordinator is responsible for managing and controlling traffic flows, computing the optimized time slot and channel assignment, and distributing the schedule throughout the network. IEEE 802.15.4e beaconing typically ensures that the centralized coordinator’s topology remains updated, which can help mitigate some of these limitations in real deployments. Additionally, when network changes occur, these updates are forwarded to the coordinator node through beacons and the coordinator node will decide to continue with the same schedule with minor changes or regenerate a new optimal schedule.

From a time complexity perspective, the algorithm was evaluated across various scenarios. However, scenarios involving 100 or more nodes present a challenge in the dynamic environments typical of industrial networks. Regenerating the optimal solution and applying a new schedule for scenarios with 100 or more sensor nodes requires considerable time, and while the network may sustain operations during this re-optimization period, this temporary period can lead to packet delays or losses due to queue overflows, resulting from changes in network topology and traffic patterns. Future research could explore more efficient re-optimization techniques to address these challenges.

## 7. Conclusions and Future Work

In this study, we introduced a novel QoS-aware approach utilizing an enhanced multi-objective differential evolution optimization algorithm to design an optimal TSCH schedule for heterogeneous sensor networks in industrial environments. The objective was to develop an algorithm to meet the QoS requirements of multiple concurrently operating applications within the network. Our approach addressed potential collisions and interference to achieve a high PDR. We implemented a dynamic pool concept to manage nodes with packets ready for transmission, therefore minimizing redundant scheduling and reducing delays, which can adversely affect PDR values.

Validation through a Matlab and TSCH-SIM co-simulation framework confirmed that the schedule generated by the QMDE algorithm effectively satisfies the QoS demands for diverse application flows. However, achieving the desired delay proved challenging in networks with 100 or more nodes due to constraints in scheduling transmissions within single time slots with a fixed number of channel offsets.

A comparison of delay and PDR between QMDE and TASA for selected applications according to ISA SP100 (closed-loop supervisory control and condition monitoring) across various scenarios revealed that QMDE consistently achieved lower delay and met QoS targets better than TASA. QMDE’s iterative optimization approach aimed to identify optimal time slots for assigning transmissions to meet targeted delay and PDR requirements. Although TASA exhibited notable PDR and low delay in scenarios with fewer nodes (16 and 36), it struggled as the network traffic and node count increased. This limitation stemmed from TASA’s challenge in optimizing time slot allocations and node assignments within TSCH schedules to consistently meet delay and PDR requirements.

In our future research, we will explore methods to enhance the effectiveness of the QMDE algorithm for computing and updating schedules in response to dynamic network changes, including topology changes, node additions, removals, and other events. This investigation aims to enhance the algorithm’s adaptability and efficiency in maintaining optimal scheduling strategies among evolving network conditions. Additionally, we plan to investigate the impact of increasing the number of channel offsets in scenarios involving large networks, aiming to evaluate how effectively this strategy reduces latency in large networks experiencing high traffic loads.

## Figures and Tables

**Table 1:** List of parameters for QMDE algorithm.

Parameter	Description	Value
*N*	Total number of nodes	16, 36, 64, 100
$S_{i}$	Sensor with ID *i*	i∈[1,…,N]
PR	Packet rate	L (1 pkt/min), M (60 pkt/min)
*R*	Communication range of node	30 m
$Dist_{Nbr}$	Distance between neighbors	20 m
*m*	Number of channel offsets	4
*n*	Maximum slotframe size	500
MaxIter	Maximum number of iteration	50
$n_{Pop}$	Number of population	5
$Var_{min}$	Lower bound of decision variables	1
$Var_{max}$	Upper bound of decision variables	Variable
$P_{CR}$	Crossover probability	0.7
$beta_{min}$	Lower bound of scaling factor	0.2
$beta_{max}$	Upper bound of scaling factor	0.8
$TD_{1}$	Target delay for App 1	50 ms
$TL_{1}$	Target packet loss for App 1	$10^{-7}$
$TD_{2}$	Target delay for App 2	100 ms
$TL_{2}$	Target packet loss for App 2	$10^{-6}$

**Table 2:** Application’s specifications.

Application	Class	Target Delay	Target Packet Loss	Packet Rate
App 1	Class 2: closed-loop supervisory control	<50 ms	<$10^{-7}$	M
App 2	Class 4: condition monitoring	<100 ms	<$10^{-6}$	L

**Table 3:** Scenario specifications.

Scenario	N	App 1 Proportion	App 2 Proportion
Scn 1	16, 36, 64, 100	M (50%)	L (50%)
Scn 2	16, 36, 64, 100	M (60%)	L (40%)
Scn 3	16, 36, 64, 100	M (70%)	L (30%)
Scn 4	16, 36, 64, 100	M (80%)	L (20%)
Scn 5	16, 36, 64, 100	M (90%)	L (10%)

**Table 4:** Simulation parameters used in TSCH-SIM simulation.

Parameter	Value
SIMULATION_DURATION	3000 s
APP_WARMUP_PERIOD_SECOND	1500 s
LINK_MODEL	Logistic Loss
APP_PACKET_SIZE	100
MAC_MAX_RETRIES	7
MAC_QUEUE_SIZE	10
LOGISTICLOSS_TRANSMIT_RANGE_M	30 m
LOGLOSS_PATH_LOSS_EXPONENT	3
TSCH_SCHEDULE_DEFAULT_LENGTH	Derived slotframe size
ROUTING_ALGORITHM	ManualRouting [35]
SCHEDULING_ALGORITHM	ManualScheduler [35]

## Data Availability

Data are contained within the article.

## Abbreviations

The following abbreviations are used in this manuscript:

ASN	Absolute Slot Number
AMUS	Adaptive Multi-hop Scheduling
CDE	Customized Differential Evolution
CMAB	Combinatorial Multiarmed Bandit
DE	Differential Evolution
EP	Expected packet
FDMA	Frequency Division Multiple Access
LI	Local Iteration
LLR	Linear Learning Rewards
MAC	Medium Access Control
MP	Matching Pairs
MST	Minimum Spanning Tree
OSCAR	Optimized Scheduling Cell Allocation Algorithm
PCDE	Priority-based Customized Differential Evolution
PDR	Packet Delivery Ratio
PR	Packet rate
QoS	Quality of Service
TASA	Traffic-Aware Scheduling Algorithm
TDMA	Time Division Multiple Access
TSCH	Time Slotted Channel Hopping
IoT	Internet of Things
WSN	Wireless Sensor Network
